# Breast-Milk Iodine Concentrations, Iodine Status, and Thyroid Function of Breastfed Infants Aged 2-4 Months and Their Mothers Residing in a South African Township

**DOI:** 10.4274/jcrpe.2720

**Published:** 2016-12-01

**Authors:** Jennifer Osei, Maria Andersson, Olivia van der Reijden, Susanne Dold, Cornelius M. Smuts, Jeannine Baumgartner

**Affiliations:** 1 North-West University, Centre of Excellence for Nutrition, Potchefstroom, South Africa; 2 ETH Zurich, Human Nutrition Laboratory, Institute of Food, Nutrition, and Health, Zurich, Switzerland

**Keywords:** Breast-milk iodine concentration, urinary iodine concentration, salt iodine concentration, lactating women, infants, thyroid hormones

## Abstract

**Objective:**

Lactating women and their infants are susceptible to iodine deficiency and iodine excess. In South Africa, no data exist on the iodine status and thyroid function of these vulnerable groups.

**Methods:**

In a cross-sectional study, urinary iodine concentrations (UIC), thyroid function, and breast-milk iodine concentrations (BMIC) were assessed in 100 lactating women from a South African township and their 2-4-month-old breastfed infants. Potential predictors of UIC, thyroid function, and BMIC, including household salt iodine concentrations (SIC) and maternal sodium excretion, were also investigated.

**Results:**

The median (25^th^-75^th^ percentile) UIC was 373 (202-627) μg/L in infants and 118 (67-179) μg/L in mothers. Median household SIC was 44 (27-63) ppm. Household SIC and maternal urinary sodium excretion predicted UIC of lactating mothers. Median BMIC was 179 (126-269) μg/L. Age of infants, SIC, and maternal UIC predicted BMIC. In turn, infant age and BMIC predicted UIC of infants. Forty-two percent of SIC values were within the South African recommended salt iodine fortification level at production of 35-65 ppm, whilst 21% of SIC were >65 ppm. Thyroid-stimulating hormone, total thyroxine, and thyroglobulin concentrations in the dried whole blood spot specimens from the infants were 1.3 (0.8-1.9) mU/L, 128±33 mmol/L, and 77.1 (56.3-105.7) μg/L, respectively, and did not correlate with infant UIC or BMIC.

**Conclusion:**

Our results suggest that the salt fortification program in South Africa provides adequate iodine to lactating women and indirectly to their infants via breast milk. However, monitoring of salt iodine content of the mandatory salt iodization program in South Africa is important to avoid over-iodization of salt.

**WHAT IS ALREADY KNOWN ON THIS TOPIC?**

South African school children and women of reproductive age have adequate iodine intake. However, more recent data point gaps in iodine nutrition of South Africans, as more than a third of the population still lacks access to adequately iodized salt. Furthermore, no data exist on iodine status in lactating women and infants. Breast-milk iodine concentrations are dependent on maternal iodine intake.

**WHAT THIS STUDY ADDS?**

This is the first study to report iodine status, breast-milk iodine concentrations (BMIC), and thyroid function of breastfed infants and lactating mothers in South Africa. Iodine in household salt (SIC) and maternal urinary iodine concentration (UIC) were predictors of BMIC, which in turn predicted the UIC of infants. Our results indicate a successful universal salt iodization program in South Africa providing adequate iodine for infants via breast milk. However, fortification of salt needs to be monitored, to avoid over-iodization of salt.

## INTRODUCTION

Dietary iodine is an essential substrate for thyroid hormone [thyroxine (T_4_) and triiodothyronine (T_3_)] synthesis and is as such required for normal brain development, growth, and metabolism (1). Both low and high iodine intake can lead to thyroid dysfunction (2). Infants may be particularly vulnerable to iodine deficiency and iodine excess as the fetal and newborn thyroid has limited iodine stores and adapts poorly to high intakes (3,4,5). Acute iodine excess from for example maternal iodine supplements and iodine containing skin disinfectants may cause hypothyroidism in newborns (6,7). Recent data indicate that older infants may be able to adapt to high iodine intakes and maintain their euthyroid state (8). However, little is known about the effects of habitual high iodine intake on thyroid function in breastfed infants.

Programs of universal salt iodization have made remarkable progress in improving iodine status worldwide, but in a handful of countries, salt iodine fortification is poorly monitored and the iodine intake is excessive (1,5).

In South Africa, in order to achieve a level of 30 ppm at retail and 15 ppm in households, iodization of table salt at a concentration of 35-65 ppm at the point of production was revised in 2006/2007 (9,10). The legislation does not involve fortification of agricultural salt or salt for processed foods. The introduction of universal salt iodization remarkably improved the iodine status of school children and of women of reproductive age. The 2005 South African Food-Based National Food Consumption Survey (NFCS-FB-I) reported a median urinary iodine concentration (UIC) in South African school children and women of reproductive age of 215 μg/L and 177 μg/L, respectively, indicating overall adequate iodine intake (11). However, more recent data point gaps in iodine nutrition of South Africans, as more than a third of the population still lacks access to adequately iodized salt (9,12). Furthermore, no data exist on iodine status in lactating women and infants.

The iodine requirements as recommended by World Health Organization (WHO) increase to 250 µg during lactation: additional to the recommended daily intake of 150 µg iodine for women of reproductive age, lactating women should consume 100 µg/day extra in order to cover the additional iodine need of their breastfed infants (10). Breastfed infants depend on iodine from breast milk for the synthesis of thyroid hormones and to build up intra-thyroidal iodine stores (13,14). Breast-milk iodine concentrations (BMIC) are determined by the maternal iodine intake; population medians have been shown to range from 9-32 µg/L in iodine deficient goitrous areas to 146 µg/L in iodine sufficient Chinese women (15,16). The WHO recommends a dietary iodine intake of 90 µg/day for infants aged 0-6 months (10).

Population iodine status is assessed by UIC, as 90% of ingested iodine is excreted through the renal system and median spot UIC directly reflects recent dietary iodine intake (1,10). In lactating women and in children <2 years, a median UIC <100 μg/L indicates insufficient iodine intake (10).

Measurement of serum or dried blood spot thyroglobulin (Tg) can be an additional useful biomarker of iodine status to accompany UIC measurements. Zimmermann et al, (17) showed that Tg is a sensitive marker for both low and high iodine intakes in school-aged children. Tg is also a sensitive indicator for iodine deficiency in adults (18,19).

Despite the importance of adequate iodine status and thyroid health in lactating women and their breastfed infants, to date, no data exist on BMIC or iodine status of infants and lactating South African women. This study therefore assessed iodine status, BMIC, and thyroid function of breastfed infants and their lactating mothers living in a township located in the North-West Province of South Africa and further explored potential predictors of UIC, thyroid function, and BMIC.

## METHODS

### Participants

This study included a convenient sample of 100 apparently healthy infants aged 2-4 months and their lactating mothers residing in two peri-urban settlements (Ikageng and Promosa) on the fringes of Potchefstroom in the Kenneth Kaunda District municipal area, in the North West Province of South Africa. The majority of residents in these townships are of Black African descent, the socio-economic status is low, and unemployment is high. Recruitment of mother-infant pairs was done at local health clinics in Ikageng and Promosa. Infants included in the study were: 1) generally healthy; 2) singletons; 3) had no history of thyroid disease; 4) currently being breastfed; and 5) not using any iodine containing supplements. Mothers included in the study were: 1) generally healthy; 2) had no history of thyroid disease; 3) currently breastfeeding; and 4) not using any iodine-containing supplements.

This study was conducted according to the guidelines laid down in the Declaration of Helsinki, and all procedures involving human subjects were approved by the Health Research Ethics Committee of the North-West University (NWU-00016-13-A1). Permission was also granted from the Provincial and District Health Departments in the North West Province to recruit mother-infant pairs for this study at local health clinics. The study protocol was fully explained by a trained study assistant fluent in the local language (Setswana or Afrikaans) and written informed consent was obtained from participating women.

### Data Collection

The study design was cross-sectional. Lactating mothers and their infants were invited to the metabolic clinic at the North-West University, South Africa, where the study procedures were conducted between 08:00 am and 12:00 am. Mothers were asked to bring samples of salt (10 g) and water (10 mL) from their homes. Upon arrival, the study protocol was fully explained to the mothers in their home language (Setswana or Afrikaans) and they signed informed consent. A detailed questionnaire was used to collect information on socio-economic characteristics, use of iodized salt, consumption of iodine-containing foods, use of iodine-containing supplements (currently and during pregnancy), and breastfeeding practices. Breastfeeding practices were divided into three categories, namely: 1) Exclusive breastfeeding; 2) Predominantly breastfeeding; 3) Partial breastfeeding (20).

Height and weight of mothers and weight, length, and head circumference of infants were measured using standard anthropometric techniques (21). For the measurements, mothers removed their shoes, emptied their pockets, and wore minimal clothing. Height measurements were done using a rigid stadiometer and recorded to the nearest 0.5 cm. Weights of the women were measured on a high capacity electronic flat scale (seca 813; Germany) and recorded to the nearest 0.1 kg. Measurements of infants were done using an infant scale (seca 334; Germany) to the nearest 2 g with no clothing or nappy. To measure length, infants wore only their nappy, and measurements were taken to the nearest centimeter on a ShorrBoard portable height-length measuring board with auto-lock sliding foot piece (Weigh and measure, LLC; USA). Head circumference was also measured using a head circumference measuring tape for infants (seca 212; Germany) to the nearest centimeter. Body mass index-for-age z-scores (BAZ) were calculated using the WHO (2006) growth standards. Wasting was defined as BAZ <-2, normal weight as BAZ ≥-2 and ≤2, risk for overweight as BAZ >1, and overweight as BAZ >2 (22).

A standard breakfast was served to the mothers at arrival at the metabolic clinic and before collection of biological samples. Spot urine samples (5 mL) were obtained from the mothers (within a maximum interval of 30 minutes after breakfast consumption), aliquoted, and stored at -80 0C until analysis. Breast milk samples (5 mL of foremilk) were obtained by manual expression. To obtain foremilk, mothers were asked to express milk from the breast that was not used at the last feed. The baby was then allowed to suckle the breast until fully satisfied. Breast milk samples were aliquoted and stored at -20 0C until analysis. Spot urine samples were collected from infants using a urine collection pad (SteriSets Uricol Set), aliquoted, and stored at -80 0C until analysis. Whole blood obtained via venipuncture or foot prick was spotted onto filter paper (Whatman 903; GE Healthcare) and allowed to dry at room temperature for 24 hours. They were then stored at -20 0C in sealed low-density polyethylene bags containing desiccant packets until analysis of thyroid hormones.

### Laboratory Analysis

UIC in spot urine samples from infants and mothers was measured in duplicate at the North-West University in Potchefstroom using the Pino modification of the Sandell-Kolthoff reaction with spectrophotometric detection (10,23). The laboratory successfully participates in the Program to Ensure the Quality of Urinary Iodine Procedures (EQUIP, U.S. Centers for Disease Control and Prevention, Atlanta GA, USA) (24). Iodine in spot urine samples from infants and lactating mothers were expressed as median concentrations (μg/L). A median UIC greater than 100 µg/L was considered to indicate adequate iodine intake in lactating women and infants (10).

In lactating mothers, we additionally determined the iodine:creatinine ratio (μg iodine/g creatinine) to reduce the intra-individual variation in daily urine volume and also to adjust for fluid intake (25,26). Urinary creatinine and sodium concentrations in spot urine from mothers were analyzed using the UniCel® DxC800 System (Beckman Coulter) at a commercial pathological laboratory (Ampath Johannesburg).

BMIC was analyzed at the Laboratory of Human Nutrition of ETH Zurich, Switzerland (27). Iodine was extracted from the samples using a modified tetramethylammonium hydroxide (TMAH) extraction procedure (28,29). The iodine content in filtered TMAH extracts was measured using a multicollector inductively coupled plasma mass spectrometer (MC-ICP-MS [Finnigan NEPTUNE, Thermo Scientific™ Waltham, MA, USA]). Quantification was done using isotope dilution analysis with 129I (SRM 4949C, National Institute of Standards and Technology [NIST], Gaithersburg, MD, USA). Tellurium (AppliChem, Darmstadt, Germany) was used for mass bias correction of the measured 127I/129I intensity ratio according to Russell’s law. The iodine concentrations of the milk samples were calculated using the dilution factors applied to each milk sample. Standard reference material (SRM 1549a, Whole Milk Powder, NIST, Gaithersburg, MD, USA) was analyzed as external control with each ICP-MS run (30). The method was recently validated at the Human Nutrition Laboratory of ETH Zurich, Switzerland. The mean [± standard deviation (SD)] iodine content for the NIST SRM1549a reference material was 3502 (±89) ng/g (n=16), well within the certified acceptable range (3040-3640 ng/g). The total-assay variability of the method is 2.6%. The within-assay variability is 1.1% and the between-assay variability is 1.3%. The limit of detection of the method is 0.26 ng/g.

Dried whole blood spots were analyzed for thyroid-stimulating hormone (TSH) (DELFIA NeoTSH kit, PerkinElmer Life Sciences, Turku, Finland) and total thyroxine (TT_4_) (Delfia Neonatal T_4_ kit, PerkinElmer Life Sciences, Turku, Finland) using automated fluoroimmunoassay (31). Analysis of dried blood spots-Tg (DBS-Tg) was done by a new sandwich enzyme-linked immunosorbent assay that was recently developed and validated at the Human Nutrition Laboratory of ETH Zurich, Switzerland (32). Serum control samples (Liquicheck Tumor Marker Control, Bio-Rad, Hercules, CA, USA) were used as standards for the DBS-Tg assay. Normal reference ranges for TSH and T_4_ as supplied by the manufacturer were as follows: TSH of 0.1-4.5 mU/L and 0.1-3.7 mU/L for 60-155-day-old infants and for subjects 1-99 years of age, respectively; TT_4_ of 80-165 nmol/L and 65-165 nmol/L for 60-155-day-old infants and for subjects 1-99 years of age, respectively. Normal range reference values for DBS-Tg are only available for school-age children (4-40 μg/L) but not for young children and infants (33,34).

Salt iodine concentrations (SIC) and water iodine concentrations (WIC) were determined by using the Pino modification of the Sandell-Kolthoff reaction with spectrophotometric detection (23). Household SIC was expressed as median and classified into three categories, according to ranges in ppm based on the 2006/2007 South African mandatory fortification level for table salt at the point of production (9). Salt samples were considered inadequately, adequately, and over-iodized when SIC was <35 ppm, 35-65 ppm, and >65 ppm, respectively. Household SIC were also classified into the three fortification categories indicating inadequately (SIC <15 ppm), adequately (SIC 15-40 ppm), or excessively (SIC >40 ppm) iodized salt at household level as recommended by WHO (35).

Data on the intake of potentially iodine-rich foods in lactating mothers were collected using an unquantified food frequency questionnaire and presented as the number (%) of mothers who consumed specific iodine-rich foods.

### Statistical Analysis

All data processing and analysis were done using IBM SPSS statistics version 20. Data were checked for normality using Q-Q plots and the Shapiro-Wilk test. Normally distributed data were presented as mean ± SD. Non-normally distributed data were presented as median (25th-75th percentiles) values. For between-group comparisons, the Mann-Whitney U or Kruskal-Wallis tests were used for non-parametric data. Spearman correlations were performed to determine associations between variables. Multiple linear regression analyses were used to explore whether household SIC, salt intake of mothers (urinary sodium excretion), UIC of mothers (only for BMIC and UIC of infants as dependent variable), age of mothers and infants, and BMIC (only for UIC of mothers and infants as dependent variables) were predictors of BMIC and UIC in lactating mothers and breastfed infants. Other dietary and maternal factors [e.g. mode of breastfeeding and delivery, smoking habits, human immunodeficiency virus (HIV) status] were also tested using a stepwise procedure, but none of those were significant predictors of BMIC or UIC of mothers and infants and were therefore not included in the final regression models. Non-parametric dependent variables were transformed prior to analysis. Furthermore, we examined the odds ratios (OR) for infants to have abnormal thyroid hormone concentrations with excessive or inadequate iodine intake using binary logistic regression analyses, adjusting for age of mothers and infants, as well as HIV status of mothers. A p-value <0.05 was considered significant.

## RESULTS

One hundred mother-infant pairs participated in the study. Characteristics of the infants and mothers are shown in Table 1. Infants were aged 3-4 months (mean ± SD: 3.0±1.1); 54 were females and 46 were males. Of all the infants, 67% were exclusively breastfed, whilst 9% and 24% were predominantly and partially breastfed, respectively.

**Figure 1:**
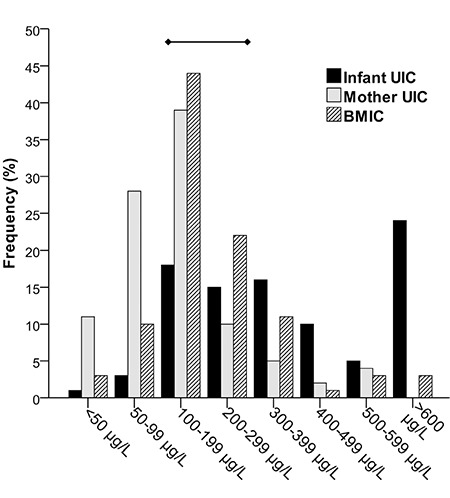
Frequency distribution of spot urinary iodine concentrations of lactating mothers (n=100) and their breastfed infants (n=92), and breast-milk iodine concentrations in µg/L. The urinary iodine concentrations range indicating sufficient iodine intake in lactating women and infants is highlighted UIC: urinary iodine concentration, BMIC: breast-milk iodine concentration

**Figure 2:**
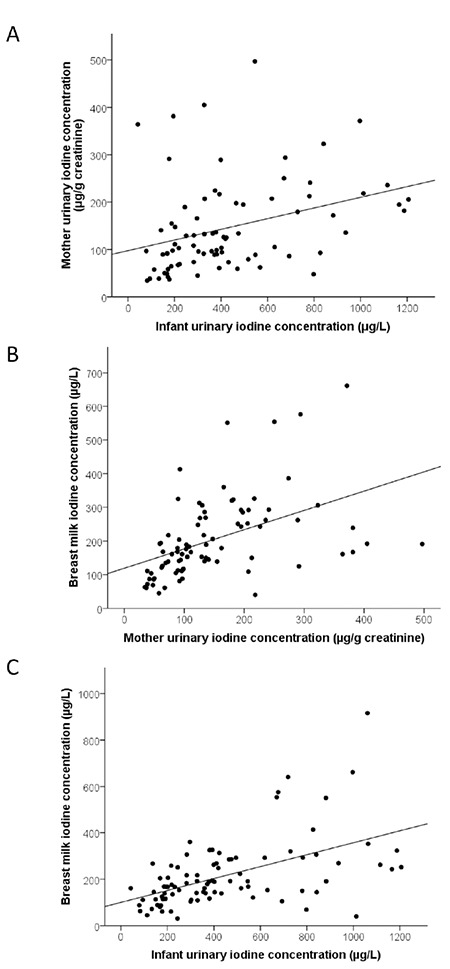
Scatter plots indicating the Spearman correlations between (A) urinary iodine concentrations of South African lactating mothers and their breastfed infants (Spearman correlations: rs=0.425 and p<0.001); (B) Urinary iodine concentrations of lactating mothers and breast-milk iodine concentrations (rs=0.593 and p<0.001); (C) Urinary iodine concentrations of infants and breast-milk iodine concentrations (rs=0.552 and p<0.001)

The median (25^th^-75^th^ percentiles) UIC of infants (n=92) was 373 (202-627) μg/L (Table 2). The median UIC of mothers was 118 (67-179) μg/L and the iodine:creatinine ratio was 126 (86-207) μg/g. Figure 1 illustrates the frequency distribution of infant and maternal spot UIC, and BMIC. Thirty-nine percent of mothers had UIC <100 μg/L, whereas, only 4% of infants had UIC <100 μg/L. Fifty-three per cent of infants had a UIC >300 μg/L and 26.1% >600 μg/L. UIC of mothers were positively correlated with the UIC of infants (r_s_=0.425, p<0.001) (Figure 2A). The UIC of both infants and mothers were positively correlated with BMIC (infants: r_s_=0.552, p<0.001; mothers: r_s_=0.593, p<0.001) (Figure 2B, 2C). Infants of obese mothers had higher UIC [495.3 (141.8-1060.9) μg/L; p=0.04] than infants of mothers that were normal weight (n=39). We found no other association of UIC in infants and mothers with participant characteristics and frequency of iodine-containing foods consumed by mothers. Generally, cow’s milk was consumed either every day or sometimes by 82% of mothers. Whilst 60% consumed fish sometimes, 69% consumed meat always (Table 3).

Median BMIC was 179 (126-269) μg/L. Median SIC (n=85) was 44 (27-63) ppm. The majority of women (90%) used adequately iodized salt in the household (≥15 ppm) as defined by WHO, 42% consumed salt that was within the South African recommended salt iodine fortification level at production (35-65 ppm), whilst 21% of households consumed salt that was iodized above 65 ppm. Iodine in water collected from the different households was below detection limit (<10 μg/L).

The UICs of infants and their mothers were positively correlated with household SIC (infants: r_s_=0.341, p<0.001; mother: r_s_=0.252, p<0.001). The median UIC of mothers from households with SIC above 65 ppm [185 (117-411) µg/L] was higher than that of mothers from households with iodized salt containing 35-65 ppm [105 (68-163) µg/L; p=0.024] and that of mothers from households with SIC below 35 ppm [117 (62-136) µg/L; p=0.004]. Likewise, the median UIC of infants from households with SIC above 65 ppm [719 (478-911) µg/L] was higher than of infants from households with iodized salt at 35-65 ppm [346 (194-530) µg/L; p=0.006] and with SIC below 35 ppm [250 (177-411) µg/L; p<0.001].

Using multiple linear regression analysis, household SIC and maternal urinary sodium excretion significantly predicted UIC of lactating mothers (Table 4). Household SIC, maternal UIC, and age of infants significantly predicted BMIC. In turn, BMIC as well as infant age significantly predicted UIC in infants.

Thyroid hormone concentrations in lactating mothers and their infants are shown in Table 2. Infant TSH, TT^4^, and Tg concentrations were 1.3 (0.8-1.9) mU/L, 128±33 mmol/L, and 77.1 (56.3-105.7) μg/L, respectively. Mother TSH, TT_4_, and Tg concentrations were 0.8 (0.6-1.0) mU/L, 69.6±15.9 mmol/L, and 22.2 (14.4-30.7) μg/L, respectively. We found that 99% of infants had TSH concentrations within the normal range. No associations of UIC were found with Tg, TSH, and thyroid hormone concentrations in infants or mothers. However, median TSH concentrations were significantly higher in HIV-positive [0.95 (0.0-1.7) mU/L] than HIV-negative [0.7 (0.0-2.2) mU/L] mothers (p=0.021). Further, maternal TT_4_ concentrations were associated with TT_4_ concentrations of their infants (r=0.236, p=0.020). TT_4_ concentrations were significantly lower in HIV-positive (61.0±15.3 nmol/L) than HIV-negative (72.1±15.3 nmol/L) mothers (p=0.004). In turn, the odds for having low TT_4_ concentrations were significantly higher in HIV-positive (TT_4_ <65 nmol/L=63%) than HIV-negative (TT_4_ <65 nmol/L=37%) mothers (OR=2.95, 95% confidence interval: 1.11-7.90). We did not observe any significant differences in Tg concentrations by HIV status. Furthermore, the thyroid hormone status of the infants did not differ with regard to maternal HIV status.

## DISCUSSION

To our knowledge, this is the first study to report iodine status, BMIC, and thyroid function of breastfed infants and lactating mothers in South Africa. Our findings suggest adequate iodine status in both lactating women and their breastfed infants in this convenience sample. Based on a BMIC of 179 µg/L and a breast-milk consumption of 0.78 L at 3 months (36), infants consumed 140 µg iodine/day, well above the recommended daily iodine intake of 90 µg and 110 µg for infants below 6 months of age by WHO and the Institute of Medicine (IOM), respectively (10,36). WHO applies the threshold of ≥100 µg/L for the median UIC to determine iodine sufficiency in children less than 2 years of age. This cut-off sharply disagrees with the intake recommendations from both WHO and IOM. By assuming a urine volume in infants of approximately 500 mL/day and 90% bioavailability and excretion (10,36), the UIC corresponding to the recommended dietary iodine intake of 90-110 µg/day would be in the range of 160-240 µg/L. Although the median UIC in the infants in our study is more than 3 times higher than the WHO UIC threshold, it should be noted that no range for median UIC reflecting optimal iodine nutrition during infancy has been defined. Considering the small urine volume in infants, a wide UIC range is expected. Previous studies in Niger and Algeria also reported high median UICs (220 µg/L and 728 µg/L, respectively) in breastfed infants (33,37,38). Lower median UICs were observed in exclusively breastfed infants from the Boston area in the U.S. (204 µg/L) and Switzerland (82 µg/L) which are both considered iodine-sufficient populations (39,40). The scientific basis for the dietary iodine requirements during infancy is weak. The IOM intake recommendation is an Adequate Intake (AI) because there were insufficient data to establish an Estimated Average Requirement (EAR) for this age group and no upper intake level has been defined (10,36). Work is on-going to add data to this knowledge gap (ClinicalTrials.gov: Project NCT02045784).

The median Tg concentrations of 77.1 µg/L is six times higher than reported in iodine-sufficient school-aged children (17). Pediatric reference ranges for serum-Tg assays indicate physiologically elevated Tg concentrations during the first two years of life (41,42). The Tg concentrations gradually decline with age. Infant reference values are lacking for DBS-Tg as well as data on DBS-Tg in iodine-sufficient breastfed infant populations. However, the magnitude of the elevated DBS-Tg concentration in infants in our study compared to median DBS-Tg concentration observed in iodine-sufficient school children suggests that Tg production may increase in response to a marginally high iodine intake in infants. However, none of the infants had subclinical hypothyroidism, and we found no associations of infant UIC with Tg, TSH, and T_4_ concentrations, indicating a possible adaptation of the thyroid gland.

Based on median UIC, lactating women in the present study have adequate iodine status. The iodine intake in the mothers is estimated to 320 µg/day; 140 µg iodine is excreted in the breast milk and 180 µg in the urine (assuming a urine volume of 1.5 liters/day). All women have normal TSH concentrations. The high prevalence of hypothyroxinemia should be interpreted with caution; we applied the TT_4_ thresholds defined for women of reproductive age in the absence of normal reference ranges for maternal TT_4_ in breastfeeding women. Cross-sectional data suggest lower TT_4_ concentrations during early lactation (43,44). In addition, we observed higher TSH and lower TT_4_ concentration in HIV-positive women than in HIV-negative women. Abnormalities in thyroid function of HIV patients have been previously described (45,46). Most HIV-positive mothers in our study reported to be on the highly active antiretroviral therapy (HAART). Increased prevalence of sub-clinical hypothyroidism is known to occur in HIV treated patients, especially those on HAART (47,48). Madeddu et al (49) emphasized the need to sequentially check thyroid function in HIV patients on HAART after they observed elevated TSH levels in these patients as compared to naive patients and controls. This might be particularly crucial in lactating women, considering the positive associations that we observed between TT_4_ concentrations in mothers and their infants.

Confirming the influential role of maternal iodine intake on the iodine status of breastfed infants (15), we found maternal UIC to be a predictor for BMIC, which in turn was a predictor for UIC in infants. Most infants included in the present study were exclusively breastfed (67%), but some mothers reported to occasionally feed their infants with commercial infant formula or other foods (for example, maize meal porridge and commercial infant cereals). We did not observe any differences in infant UIC based on feeding practices. Our findings, however, show that infant age is a strong negative predictor for BMIC, confirming that BMIC may decline within the first six months of lactation (50). Furthermore, discrepancies in literature exist regarding the relationship between maternal and infant UIC. In agreement with our study, various authors have found positive correlations between infant and mother UIC (38,51,52), while others did not (53,54).

Our results indicate that household salt was a major source of iodine for mothers; SIC predicted both BMIC and maternal UIC. The majority (90%) of women consumed adequately iodized salt (>15 ppm) and our data indicate that the iodized salt coverage in the Potchefstroom area is high and meets the WHO criteria for a successful salt iodization program (10). In 2005, a national study reported that 77% of households in South Africa had access to and consumed adequately-iodized salt (9). The median iodine concentration in the household salt was 44 ppm, ranging from 0-153 ppm, slightly above the upper level of 40 ppm recommended by WHO (10). Iodine is a volatile micronutrient (55) and the iodine fortification level in the South African program (35-65 ppm) has been set to account for possible losses in salt iodine concentrations before salt reaches households. However, our data also show that 21% of households consumed salt iodized above the upper level of 65 ppm. It has been previously documented that poor implementation and insufficient monitoring of universal salt iodization programs worldwide have resulted in inadequate and even excess intakes of iodine in several countries (56). In South Africa, the median iodine concentration in household salt has previously been reported to range from 6 ppm to 42 ppm across all provinces and 30 ppm nationwide (57).

The daily quantity of salt consumed by mothers was beyond the scope of this study and sodium excretion was measured only in spot urine samples, which is not recommended for estimating individual sodium intake. However, 90% of mothers indicated that they used salt every day for food preparation. The South African population is known to have high salt intakes. The mean salt consumption in the country is 6-12 g per day per person, which is higher than the WHO recommendation of ≤5 g of salt (<2000 mg sodium) per day per person (58). Currently, policies are being implemented to reduce the salt intake of the general population. Salt intakes as low as 5 g per day are known to have adequate amounts of iodine, provided the salt is sufficiently iodized (12). Our results indicate that the amount of iodine added to salt by some producers may be to too high and that the compliance with the current legislation (35-65 ppm) is not properly monitored and may therefore lead to over-iodized salt in the market.

In South Africa, fortification of salt is only mandatory for table salt and not for salt used in processed foods. In this study, sodium excretion and household SIC were independent predictors of maternal UIC, indicating that iodized household salt may not have been the sole source for iodine. Thus, the possibility of obtaining iodine from other food sources cannot be over-ruled. For example, there was a reported frequent consumption of cow’s milk, and the assessment of various brands of milk available in the local market showed that the iodine content in milk ranged from 116-366 μg/L (unpublished results). Only few studies have determined whether processed foods in South Africa contain iodized salt. Bread is the major source of dietary salt intake for adults, especially urban black dwellers, who are said to obtain 49-54% of their salt intake from bread and cereal food groups (59). Several manufacturing companies have previously reported to use salt containing substantial amounts of iodine (39-69 ppm), especially for food items that were frequently distributed countrywide (60). These reports are, however, old and it is likely that more food manufacturing companies now use iodized salt.

A limitation of this study is the small sample size. However, the vast amount of data collected in the study provides a complete picture of the iodine status in the studied infants and their mothers. We did not collect data on the use of any iodine containing disinfectants applied for maternal wound disinfection or continuous umbilical care of the infants and acknowledge this limitation (61). Based on information received from clinics and the local hospital, the most commonly used disinfectant in theatre during caesarean delivery is HibiTane^®^ (containing chlorhexidine) in either alcohol or iodine, and water in chlorhexidine is also being used for perineal laceration. Iodine is preferably used over alcohol as it is believed to cause less irritation. However, we did not observe any significant difference in UIC between infants born via vaginal delivery or caesarean, therefore ruling out possible contamination from maternal wound disinfectants. Furthermore, in clinics, mothers are mainly advised to use alcohol (surgical spirit) for umbilical care and hence iodine contamination is unlikely.

In conclusion, our results suggest that iodized salt is a major contributor to iodine status in lactating mothers and their infants. The results also show that the salt iodization program in South Africa not only supplies sufficient iodine for children and women of reproductive age, but also for lactating mothers and breastfed infants. However, salt iodine levels appear to be poorly monitored. There is a dire need for on-going monitoring and surveillance of salt fortification at production, to avoid over-iodized salt and ensure sustenance of optimal iodine status in vulnerable population groups.

## Acknowledgments

The authors would like to thank all participating mothers and the participating clinics from the Potchefstroom municipality. Our gratitude is also extended to the manager of the metabolic unit, Chrissie Lessing, for making this study possible and ensuring it was well organized. We also thank all the nurses and fieldworkers who assisted with the study procedures. This study was funded by the Human Nutrition Laboratory of ETH Zurich, Switzerland, the Nestle Nutrition Institute Africa and the South African Sugar Association.

## Ethics

Ethics Committee Approval: This study was conducted according to the guidelines laid down in the Declaration of Helsinki and all procedures involving human subjects were approved by the Health Research Ethics Committee (HREC) of the North-West University (NWU-00016-13-A1), Informed Consent: Permission was also granted from the Provincial and District Health Departments in the North West Province to recruit mother-infant pairs for this study at local health clinics.

Peer-review: Externally peer-reviewed.

## Figures and Tables

**Table 1:**
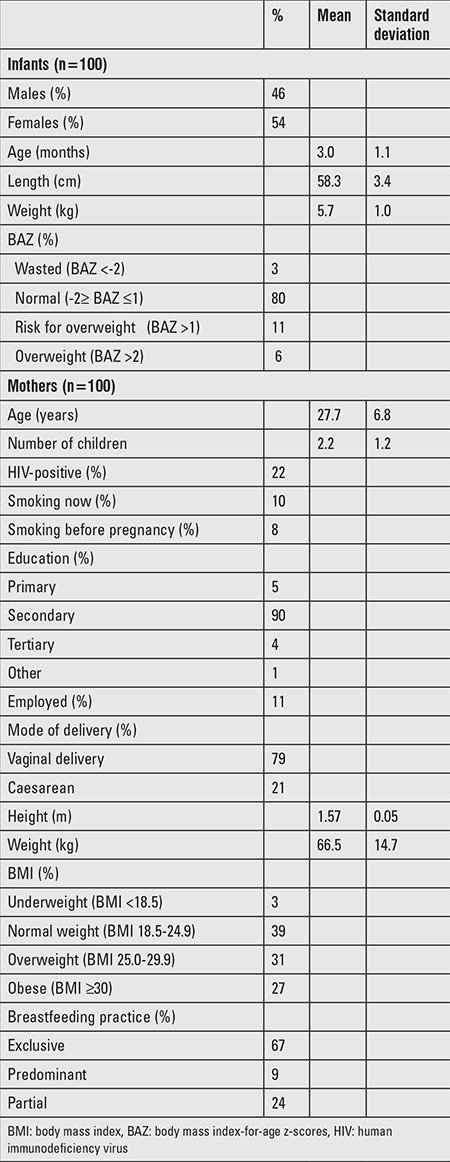
Characteristics of breastfed infants and their lactating mothers

**Table 2:**
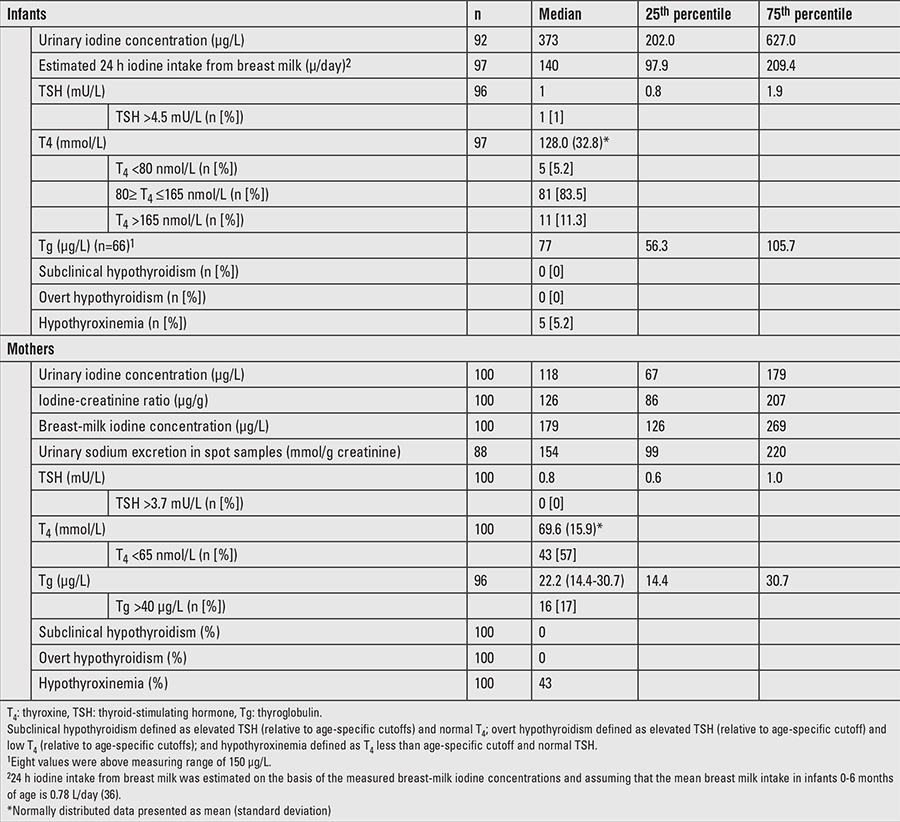
Urinary iodine and thyroid hormone concentrations in South African lactating mothers and breastfed infants

**Table 3:**
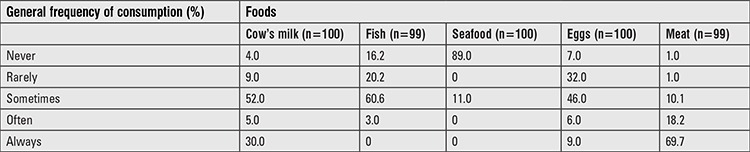
Consumption patterns of iodine-containing foods

**Table 4:**
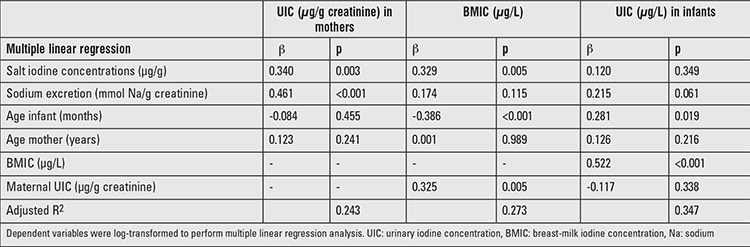
Predictors of breast-milk iodine concentration and urinary iodine concentration in South African lactating mothers and breastfed infants
